# Tumour necrosis factor- **α** and transforming growth factor- **β** are significantly associated with better prognosis in non-small cell lung carcinoma: putative relation with *BCL* -2-mediated neovascularization

**DOI:** 10.1054/bjoc.2000.1345

**Published:** 2000-07-24

**Authors:** L Boldrini, A Calcinai, E Samaritani, F Pistolesi, A Mussi, M Lucchi, C A Angeletti, F Basolo, G Fontanini

**Affiliations:** Department of Oncology, Division of Pathology, Department of Cardio-Thoracic Surgery, University of Pisa, Pisa, Italy

**Keywords:** NSCLC, tumour necrosis factor alpha, transforming growth factor beta, angiogenesis, prognosis

## Abstract

Recent in vivo and in vitro studies have demonstrated a wide spectrum of biologic activities of cytokines in the pathogenesis and progression of malignancy. Tumour necrosis factor alpha (TNF-α) and transforming growth factor beta (TGF-β) have emerged as two of the many host-derived mediators that seem to interfere with both antiproliferative and tumorigenic effects in malignant tumours including lung cancer. However, their association with tumour prognosis or prognostic factors has not yet been completely clarified. In this study, we assessed TNF-α and TGF-β mRNA expression by RT-PCR technique in 61 NSCLC samples, demonstrating the presence of TNF-α and TGF-β mRNA in 55.74% and 45.9% of cases, respectively. We also evaluated the expression of the two distinct transmembrane TNF receptors. TNFR-I and TNFR-II, with a PCR-positive signal in 70.49% and 65.57% of cases, respectively. In 49 of the 61 cases, we evaluated the prognostic impact of the two growth-inhibiting factors using the Kaplan–Meier analysis. In the univariate analysis patients without nodal metastatic involvement (*P* = 0.02), less advanced tumour stage (*P* = 0.02) or TNF-α and TGF-β positive cancers (*P* = 0.01 and *P* = 0.03) showed a favourable prognosis in terms of overall survival. Since our previous studies demonstrated a significant association between NSCLC behaviour, neoangiogenesis and *bcl* -2 expression, we investigated the putative relation between TNF-α and TGF-β on the one hand, and vascular count (as a measure of tumour angiogenesis) and *bcl* -2 protein expression, on the other hand. Our results showed a significant direct association between TNF-α and *bcl* -2 (*P* = 0.05) and an inverse association between TNF-α and microvessel count (*P* = 0.03). Moreover, as previously demonstrated, we observed a significant inverse correlation between *bcl* -2 protein expression and vascular count (*P* = 0.05), suggesting that the favourable effect of TNF-α on clinical outcome may be related to a *bcl* -2-mediated low neovascular development. © 2000 Cancer Research Campaign


*British Journal of Cancer* (2000) **83**(4), 480–486
© 2000 Cancer Research Campaign
doi: 10.1054/ bjoc.2000.1345, available online at http://www.idealibrary.com on IDEAL®

### Tumour necrosis factor-α and transforming growth factor-β are significantly associated with better prognosis in non-small cell lung carcinoma: putative relation with *BCL*-2-mediated neovascularization

**L Boldrini[1], A Calcinai[1], E Samaritani[1], F Pistolesi[1], A Mussi[2], M Lucchi[2], CA Angeletti[2], F Basolo[1] and G Fontanini[1]**

[1]Department of Oncology, Division of Pathology; [2]Department of Cardio-Thoracic Surgery, University of Pisa, Pisa, Italy

**Summary** Recent in vivo and in vitro studies have demonstrated a wide spectrum of biologic activities of cytokines in the pathogenesis and progression of malignancy. Tumour necrosis factor alpha (TNF-α) and transforming growth factor beta (TGF-β) have emerged as two of the many host-derived mediators that seem to interfere with both antiproliferative and tumorigenic effects in malignant tumours including lung cancer. However, their association with tumour prognosis or prognostic factors has not yet been completely clarified. In this study, we assessed TNF-α and TGF-β mRNA expression by RT-PCR technique in 61 NSCLC samples, demonstrating the presence of TNF-α and TGF-β mRNA in 55.74% and 45.9% of cases, respectively. We also evaluated the expression of the two distinct transmembrane TNF receptors. TNFR-I and TNFR-II, with a PCR-positive signal in 70.49% and 65.57% of cases, respectively. In 49 of the 61 cases, we evaluated the prognostic impact of the two growth-inhibiting factors using the Kaplan–Meier analysis. In the univariate analysis patients without nodal metastatic involvement ($P = 0.02$), less advanced tumour stage ($P = 0.02$) or TNF-α and TGF-β positive cancers ($P = 0.01$ and $P = 0.03$) showed a favourable prognosis in terms of overall survival. Since our previous studies demonstrated a significant association between NSCLC behaviour, neoangiogenesis and *bcl*-2 expression, we investigated the putative relation between TNF-α and TGF-β on the one hand, and vascular count (as a measure of tumour angiogenesis) and *bcl*-2 protein expression, on the other hand. Our results showed a significant direct association between TNF-α and *bcl*-2 ($P = 0.05$) and an inverse association between TNF-α and microvessel count ($P = 0.03$). Moreover, as previously demonstrated, we observed a significant inverse correlation between *bcl*-2 protein expression and vascular count ($P = 0.05$), suggesting that the favourable effect of TNF-α on clinical outcome may be related to a *bcl*-2-mediated low neovascular development. © 2000 Cancer Research Campaign

**Keywords**: NSCLC; tumour necrosis factor alpha; transforming growth factor beta; angiogenesis; prognosis

The development of a new vascular bed from a pre-existing vascular network is an essential requirement for tumour growth and progression. This phenomenon is regulated by a complex network of cytokines, enzymes and adhesion molecules (Blood and Zetter, 1990) and recent studies have shown that lymphocytes and macrophages, as well as malignant cells, represent an important source of such angiogenic factors within the tumour microenvironment (Leek et al, 1994). Tumour necrosis factor alpha (TNF-α) is a cytokine produced by different kind of human cells (monocytes, macrophages, etc) and in particular from some tumour-associated macrophages (TAMs). Although some works have reported the endogenous expression of TNF-α in the epithelial component of tumours, such as ovarian carcinoma (Naylor et al, 1993), the majority of studies have found TNF-α expression confined to the tumour-infiltrating inflammatory cells in the tumour stroma. Indeed, lymphocytes isolated from breast cancer biopsies secrete TNF-α in vitro (Rubbert et al, 1991), and various techniques have been used to visualize the production of TNF-α mRNA (Miles et al, 1994), intracellular TNF-α protein (Pusztai et al, 1994) and secreted TNF-α by TAMs in breast carcinoma (Lewis and McGee, 1996). However, the multifaceted roles of this cytokine within solid tumours are largely unknown. Growth factors of the transforming growth factor beta family (TGF-β) have been reported as multifunctional growth factors and are synthesized by a wide variety of both normal and malignant cells (Derynk et al, 1985). TGF-β has multiple actions (Fajardo et al, 1996) and, owing to its highly potent inhibition function on cell growth (Ueki et al, 1992), many studies have evaluated the prognostic significance of the expression of this factor, especially in breast cancer (Murray et al, 1993; Auvinen et al, 1995), but only limited and confusing data are available at the moment.

Our preliminary studies have underlined that the clinical behaviour of NSCLC is strongly influenced by the development of a newly formed vascular bed (Fontanini et al, 1997) which seems to be under the control of particular proliferation-related genes such as *bcl*-2 (Fontanini et al, 1998). In this respect it would be very interesting to analyse the pattern of TNF-α and TGF-β in this type of cancer to understand better their role as prognostic indicators and to verify the putative interactions with angiogenesis and with its genetic regulators.

*Received 19 November 1999*
*Revised 10 April 2000*
*Accepted 10 April 2000*

*Correspondence to:* G Fontanini

### MATERIALS AND METHODS

### Surgical specimens

61 NSCLC patients who had undergone curative surgical resection at the Department of Surgery, University of Pisa, between 1991 and 1997, were analysed. There were 47 males and 14 females (mean age 62.9 years, median 64, range 42–77). The most common histologic type was squamous carcinoma (25 cases) followed by adenocarcinoma (17 cases), large cell anaplastic carcinoma (4 cases) and bronchiolo-alveolar carcinoma (3 cases). Tumour samples were in part frozen in liquid nitrogen and stored at –80°C for molecular studies and in part formalin-fixed and paraffin-embedded for histological and immunohistochemical processing. Data on clinical behaviour were available in 49 of the 61 cases (median follow-up 77 months). Tumours were classified according to the World Health Organization classification (1982) and according to the guidelines of the American Joint Committee for Cancer Staging (1992).

### RNA extraction

Total RNA was extracted from frozen lung-tissue samples using RNeasy Mini Kit (Quiagen, M-Medical srl, Florence, Italy); the RNeasy procedure represents a novel technology which combines the selective binding properties of a silica-gel-based membrane with the speed of the microspin principle. Biological samples are first lysed and homogenized in the presence of a highly denaturing guanidinium isothiocyanate-containing buffer which immediately inactivates RNases to ensure isolation of intact RNA. Ethanol is added to provide appropriate binding conditions and the sample is then applied to an RNeasy mini spin column where the total RNA binds to the membrane and contaminants are efficiently washed away. High-quality RNA is then eluted in 30 μl of water. Purified RNA was digested with RNase-free DNase to guarantee that RNA is completely free of DNA contamination.

### RT-PCR analysis

A constant amount of total RNA (5 μg) was reverse-transcribed at 42°C for 60 min in a total 20 μl reaction volume using 1st-strand™ cDNA Synthesis Kit (Clontech Laboratories Inc, Palo Alto, CA, USA). cDNA was incubated at 95°C for 5 min to inactivate the reverse transcriptase, and served as a template DNA for amplification using the Gene Amp PCR System 2400 (Perkin-Elmer Applied Biosystems, CA, USA). PCR was performed in a standard 50 μl reaction mixture consisting of 10 mM Tris-HCl, 50 mM KCl, 1.5 mM $MgCl_2$ (pH 8.3), 0.2 mM dNTPs, 50 pmoli of each sense and antisense primer, and 2.5 U of Amplitaq DNA Polymerase (EUROBIOTAQ ADN Polymerase, Les Ulis Cedex B, France). Amplification was performed for 45 s at 94°C, 45 s at 60°C and 2 min at 72°C for 35 cycles. Lastly, an additional extension step was performed for 7 min. As negative control the DNA template was omitted in the reaction.

PCR primers for TNF-α cDNA were as follows: forward primer, 5′-GAGTGACAAGCCTGTAGCCCATGTTGTAGCA-3′; reverse primer, 5′-GCAATGATCCCAAAGTAGACCTGCCCA-GACT-3′ (Clontech Laboratories Inc). Amplified PCR products were run on a 1.5% agarose gel containing 0.5 μg $ml^{-1}$ ethidium bromide and a single 444 bp band was detected. PCR primers for TNFR-I cDNA were as follows: forward primer, 5′-ATTTGCTG-TACCAAGTGCCACAAAGGAACC-3′; reverse primer, 5′–GTC-GATTCCCACAAACAATGGAGTAGAGC-3′ (Clontech Laboratories Inc). PCR primers for TNFR-II cDNA were as follows: forward primer, 5′-GAATACTATGACCAGACAGCTCAGAT-GTGC-3′; reverse primer, 5′-TATCCGTGGATGAAGTCGT-GTTGGAGAACG-3′ (Clontech Laboratories Inc). Amplified PCR products were run on a 1.5% agarose gel containing 0.5 μg $ml^{-1}$ ethidium bromide and a single 587 bp for TNFR-I and 403 bp for TNFR-II band was detected. PCR primers for TGF-β cDNA were as follows: forward primer, 5′-GCCTCGGACACCAAC-TATTGCT-3′; reverse primer, 5′-AGGCTCCAAATGTAGG-GGCAGG-3′ (Clontech Laboratories Inc). The presence of a PCR band amplified with primer specific for GAPDH with the same cDNAs was used as internal control. Amplified PCR products were run on a 1.5% agarose gel containing 0.5 μg $ml^{-1}$ ethidium bromide and a single 161 bp band for TGF-β was detected. No band was detected when no cDNA was added to PCR mixture.

The sequencing of the PCR products, carried out using the cyclic kit (Perkin-Elmer) according to the manufacture's recommendation, has shown that we were observing the expected fragments, in particular as concerns the fragment of TNF-α which is located in the intron III between positions 404 and 847 and the fragment of TGF-β which is located in the Intron IV between positions 1678 and 1838 (data not shown).

### Quantitative PCR reaction

In order to obtain the quantitation of TNF-α and TGF-β mRNA levels we used a technique based on a competitive PCR approach using non-homologous internal standard called PCR MIMICs (Clontech). The method, already described by Boldrini et al (1999), involves amplification of a heterologous DNA fragment (*Bam*HI/*Eco*RI 574 bp fragment of v-*erb*B) with a pair of composite primers, which contain the target primer sequences contiguous to a sequence that anneals the heterologous DNA fragment. During amplification the target primer sequences were incorporated into the products. Thus, we refer to this heterologous competitor fragment as COMPETITOR because it competes with the target gene for primer annealing and amplification. Known amounts of this COMPETITOR (600 bp for TNF-α and 240 bp for TGF-β) were added to aliquots of cDNA derived from 8 μg of total RNA. The relative densitometric measure of the electrophoretic bands was then plotted and the point of equal intensity between the bands of competitor PCR MIMIC and TSP was taken as concentration of the cDNA sample.

### Microvessel detection and counting

The method of microvessel detection and counting has been described previously (Fontanini et al, 1997). Briefly, intratumour microvessels were highlighted with anti-CD34 Mab (clone QB-END 10, Novocastra, Newcastle, UK) which was diluted 1:100, overnight. Biotinylated anti-mouse IgG (Vector, Burlingame, CA, USA) was applied for immunoreaction, followed by detection using the ABC method. A single microvessel was defined as any brown immunostained endothelial cell separated from adjacent microvessels, tumour cells and other connective tissue elements. Each sample was examined under low power (×10 objective lens and ×10 ocular lens) to identify the region of the section with the highest number of microvessels. A ×250 field (×25 objective lens and ×10 ocular lens; 0.74 $mm^2$ per field) was

© 2000 Cancer Research Campaign 

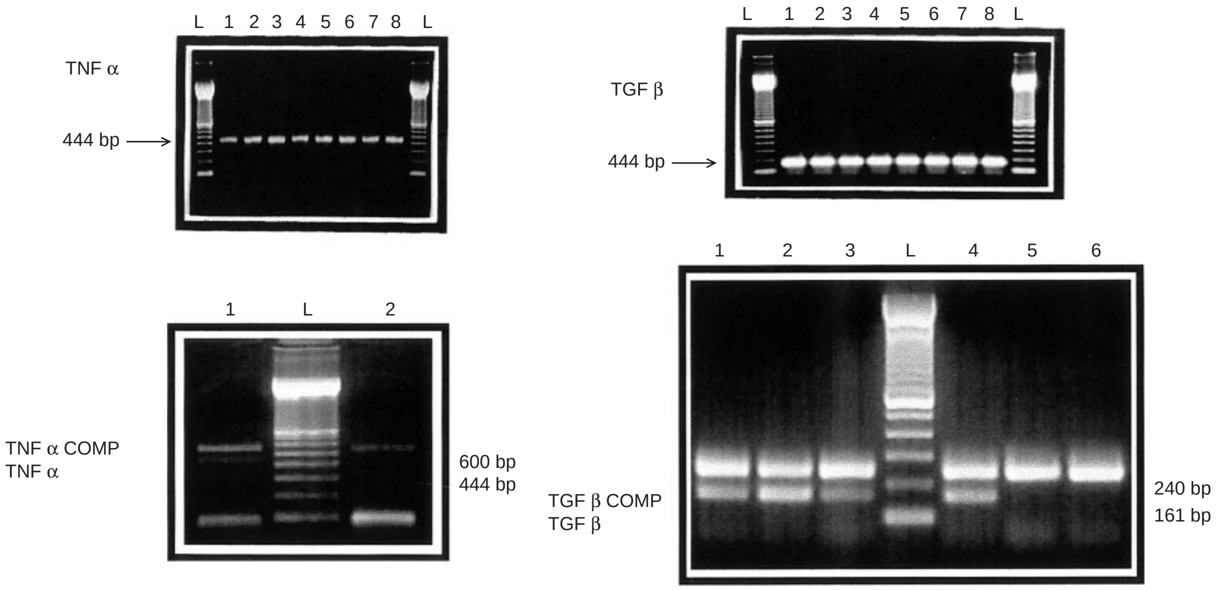

**Figure 1** Upper panel: Electrophoretic analysis of PCR products for TNF-α and TGF-β on a 1.5% agarose gel containing 0.5 μg ml$^{-1}$ ethidium bromide. A single 444 bp band for TNF-α and a single 161 bp band for TGF-β were detected. Lane L: molecular weight markers (100 bp ladder, Pharmacia). Lanes 1–7: seven different tumour samples. Lane 8: positive control. Lower panel: Electrophoretic analysis of competitive PCR products for TNF-α and TGF-β on a 1.5% agarose gel containing 0.5 μg ml$^{-1}$ ethidium bromide. The length of competitor was 600 bp for TNF-α and 240 bp for TGF-β. Lane L: molecular weight markers (100 bp ladder, Pharmacia). Lane 2 (left) and 6 (right): amplification of competitor alone; lane 5: negative sample; lanes 1 (left) and 1–4 (right): coamplification of competitor and target gene cDNA

counted in each area, and the count was recorded. Large vessels with thick muscular walls were excluded in the counts. The lumen was not required to identify a vessel.

### *Bcl*-2 immunostaining

The 5 μm tumour sections were immunostained using the alkaline-phosphatase-anti-alkaline phosphatase (APAAP) method (Cordell et al, 1984) with the anti-*bcl*-2 monoclonal antibody (clone 124) (Pezzella et al, 1992) raised to a synthetic peptide. Briefly, paraffin sections were dewaxed in xylene and rehydrated through graded alcohols. The monoclonal antibody *bcl*-2 124 was applied overnight at 1:20 of dilution. A rabbit anti-mouse secondary antibody prediluted in 0.05 M Tris-buffered and containing normal swine serum was applied for 30 min. The alkaline-phosphatase-mouse anti-alkaline-phosphatase immune complex was applied for 30 min first and then for 10 min spaced by the use of the anti-mouse serum. Reaction was developed with alkaline-phosphatase substrate containing naphthol AS-MX, Fast-red Tr and Levamisol (APAAP Kits, Dako Corporation). As positive control for *bcl*-2 we used a paraffin-embedded section from a normal peribronchial lymph-node removed during post-surgical sampling of a lung tumour. At the same time, positive staining of small lymphocytes provided internal control for *bcl*-2 staining. Staining without the anti-*bcl*-2 monoclonal antibody was performed as negative control procedure. The count of *bcl*-2 immunoreactivity was made by scoring a minimum of five high-power fields (HPFs) (×40 objective lens).

### Statistical analysis

All statistical analyses were carried out using STATISTICA software (Stat-soft). Univariate analysis was performed by modeling Kaplan–Meier survival curves. The Log-rank test was used to evaluate the statistical significance of differences in survival distributions among prognostic groups. Multivariate analysis was carried out using the Cox proportional-hazard model. Mann–Whitney and/or Kruskall–Wallis non-parametric tests were used to compare clinico-pathological characteristics of the tumours with median immunostaining values. The a priori level of significance was set at *P*-value of less than 0.05.

### RESULTS

### TNF-α and TGF-β expression

TNF-α mRNA expression evaluated by PCR technique was observed in 34 out of 61 cases (55.74%). TGF-β mRNA was detected in 28 out of 61 cases (45.9%). Figure 1 (Upper panel) shows the results of the electrophoretic analysis of PCR products in representative cases. We have also quantified the expression of the mRNA for both the cytokines using a competitive PCR technique, as described in Materials and Methods. Figure 1 (lower panel) shows the results of coamplification of COMPETITOR and target-gene cDNA in representative cases.

### TNF-receptors expression

The expression of the two distinct transmembrane TNF-receptors, TNFR-I and TNFR-II, was also studied, with a PCR-positive signal in 70.49% (43/61) and 65.57% (40/61) of cases, respectively. An example of electrophoretic gel for PCR product of TNFR-I and TNFR-II is shown in Figure 2.

© 2000 Cancer Research Campaign

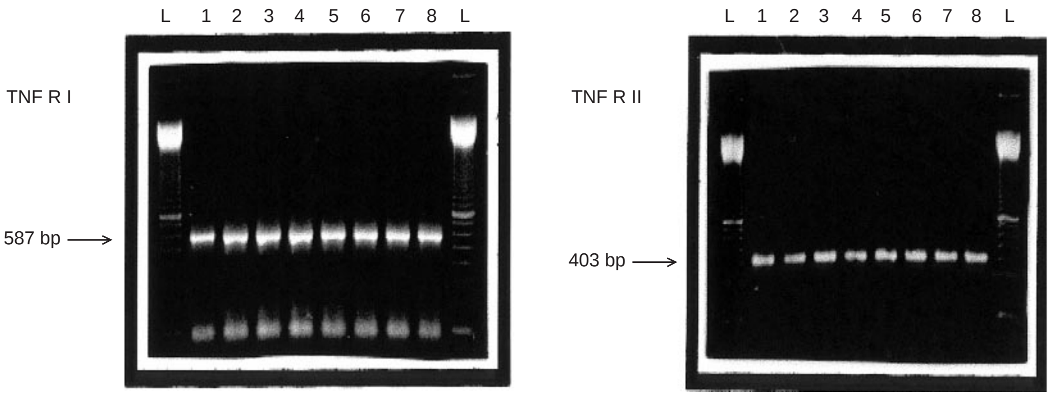

**Figure 2** Electrophoretic analysis of PCR products for TNFR-I (left panel) and TNFR-II (right panel) on a 1.5% agarose gel containing 0.5 μg $ml^{-1}$ ethidium bromide. A single 587 bp band for TNFR-I and a single 403 bp band for TNFR-II were detected. Lane L: molecular weight markers (100 bp ladder, Pharmacia). Lanes 1–7: seven different tumour samples. Lane 8: positive control

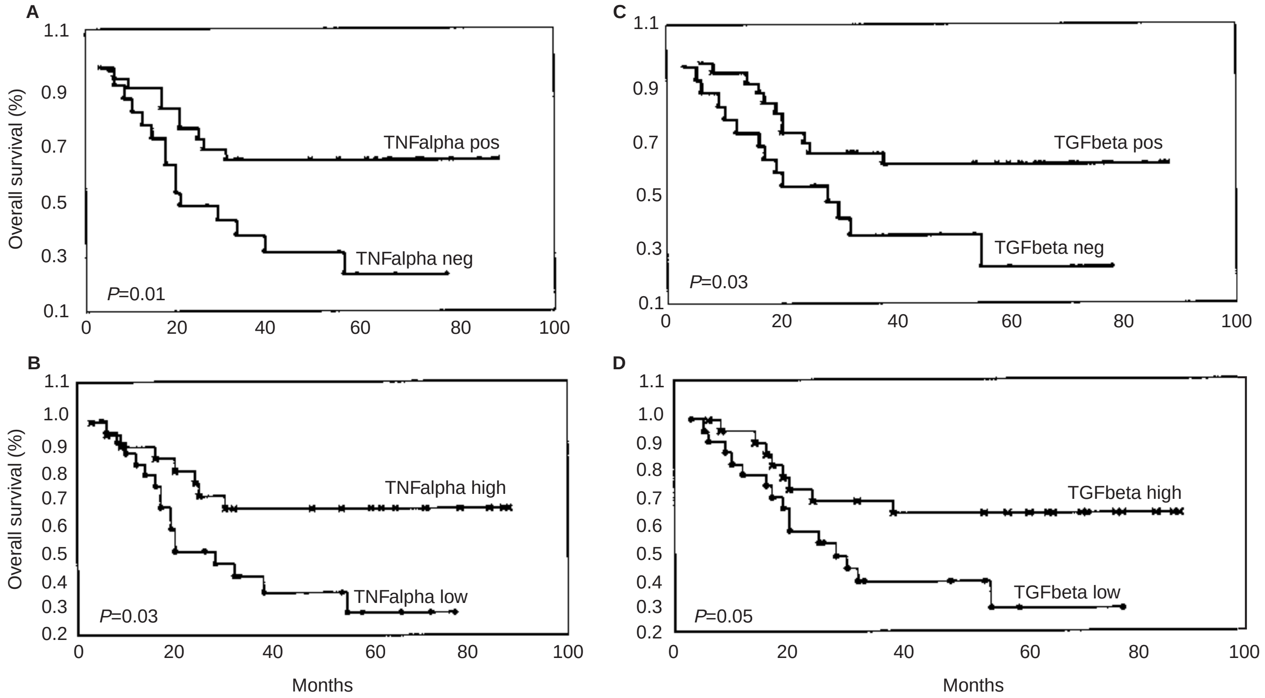

**Figure 3** Overall survival according to (**A**) TNF-α mRNA expression and (**B**) TGF-β mRNA expression, in 49 patients with non-small lung cancer (o = alive patients; x = dead patients). Overall survival curves obtained with competitive PCR for TNF-α mRNA expression (**C**) and TGF-β mRNA expression (**D**)

### Association of TNF-α and TGF-β with clinico-pathological characteristics and survival

We analysed the expression of TNF-α and TGF-β according to different clinico-pathologic parameters, including histologic type, but we did not find any significant statistical association.

Among the clinico-pathologic parameters analysed, the absence of metastatic nodal involvement and early-staged tumours were significantly associated with better overall survival ($P$ = 0.02) (Table 1). A similar statistically significant association was observed between these characteristics and disease-free interval (data not shown). At the univariate analysis TNF-α and TGF-β were significantly associated with a favourable prognosis in terms of overall survival ($P$ = 0.01 and 0.03) (Table 1) (Figure 3, A and B). Similar results were observed between TNF-α and TGF-β and disease-free interval (data not shown). By multivariate analysis nodal status, TNF-α and TGF-β expression retained an independent level of significance ($P$ = 0.02; $P$ = 0.05; $P$ = 0.05) (Table 2). On the basis of the number of the cDNA molecules valuated with competitive PCR for both TNF-α and TGF-β, we calculated the median values for the two series of the samples (47 for TNF-α and 187 for TGF-β). In this way we distinguished tumours with low from tumours with high expression of the two cytokines and we performed survival analyses by the Kaplan–Meier method (Figure 3, C and D) confirming the prognostic value for both TNF-α and TGF-β.

No correlation was observed between TNFR-I and TNFR-II expression and survival.

© 2000 Cancer Research Campaign

**Table 1** Univariate analysis

| Patient and tumour characteristics | Cases (*n*) | Two-sided *P* |
|---|---|---|
| Sex | | |
| Male | 39 | 0.34 |
| Female | 10 | |
| Histology | | |
| Squamous | 25 | 0.77 |
| Nonsquamous | 14 | |
| Tumour size | | |
| T1 | 10 | |
| T2 | 34 | 0.07 |
| T3 | 5 | |
| Node status | | |
| N0 | 32 | |
| N1 | 4 | 0.02 |
| N2 | 13 | |
| Stage | | |
| S1 | 31 | |
| S2 | 3 | 0.02 |
| S3 | 15 | |
| TNF-α | | |
| Neg | 21 | 0.01 |
| Pos | 28 | |
| TGF-β | | |
| Neg | 21 | 0.03 |
| Pos | 28 | |

**Table 2** Cox's proportional regression model of overall survival

| Variables | Beta | St. Err. of Beta | *t* | *P* |
|---|---|---|---|---|
| Gender | 0.182 | 0.279 | –0.654 | NS |
| Age | –0.461 | 0.453 | –1.018 | NS |
| Tumour status | 0.452 | 0.296 | 1.527 | NS |
| Node status | 0.775 | 0.252 | 3.111 | 0.02 |
| Stage | –0.662 | 0.486 | 1.361 | NS |
| TNF-α | –1.519 | 0.554 | –2.739 | 0.05 |
| TGF-β | –1.248 | 0.492 | –2.535 | 0.05 |

**Table 3** Relationship between TNF-α mRNA expression and *bcl*-2 protein expression in 20 cases of non-small cell lung cancer

| | | TNF-α | | |
|---|---|---|---|---|
| | | Pos | Neg | *P* |
| bcl-2 | Pos | 1 | 9 | 0.05 |
| | Neg | 5 | 5 | |

**Table 4** Relationship between TNF-α mRNA expression and MVC in 46 cases of non-small cell lung cancer

| | | TNF-α | | |
|---|---|---|---|---|
| | | Pos | Neg | *P* |
| MVC | ≤20 | 17 | 6 | 0.03 |
| | >20 | 10 | 13 | |

**Table 5** Relationship between bcl-2 protein expression and MVC in 20 cases of non-small cell lung cancer

| | | *bcl*-2 | | |
|---|---|---|---|---|
| | | Pos | Neg | *P* |
| MVC | ≤20 | 9 | 5 | 0.05 |
| | >20 | 1 | 5 | |

### Correlation with neoangiogenesis and *bcl*-2 expression

A significant direct association between TNF-α and *bcl*-2 was found (*P* = 0.05) (Table 3). Furthermore, TNF-α mRNA was inversely associated with microvessel count. In fact, tumours with a TNF-α mRNA positivity showed a significantly lower microvessel density (≤ 20 microvessels) than tumours which did not show TNF-α mRNA expression (*P* = 0.03) (Table 4). Moreover, as previously demonstrated, we observed an inverse significant correlation between *bcl*-2 protein expression and vascular count (*P* = 0.04) (Table 5).

### DISCUSSION

### TNF-α expression

In this study we analysed the expression of two important cytokines in a series of NSCLC in order to investigate their putative role in lung cancer behaviour and their relation with other biological parameters which are likely to have an important prognostic impact in this type of cancer (Pezzella et al, 1993; Silvestrini et al, 1994; Fontanini et al, 1997; Koukourakis et al, 1997). In particular neoangiogenesis and *bcl*-2 protein expression have shown to significantly affect NSCLC development and progression, and in our previous studies we also underlined the relationship between neoangiogenesis and *bcl*-2 protein (Fontanini et al, 1998). The development of newly formed tumour microvessels is regulated by a large network of interrelating factors rather than one factor alone (Lewis and Balkwill, 1997).

In experimental systems, TNF-α can both inhibit and stimulate angiogenesis in a dose-dependent manner, with high doses being inhibitory, and low doses stimulatory (Fajardo et al, 1992; Leek et al, 1994). In a number of immunohistochemical studies TNF-α has appeared in particular in the cytoplasm of inflammatory cells and tumour-associated macrophages (Pusztai et al, 1994), although a proportion of neoplastic cells have shown to be TNF-α positive in some human cancers such as breast (Leek et al, 1998) and lung carcinomas (Tran et al, 1998). Substantially different results have been obtained in these human models: in breast carcinoma a significant association has been found between TNF-α and thymidine phosphorylase expression, which is a factor known to promote tumour angiogenesis (Fajardo et al, 1992; Fox et al, 1996); in lung cancer, where neoangiogenesis seems to play an unfavourable prognostic role, TNF-α expression has shown to produce a better clinical outcome (Tran et al, 1998). In contrast, an antiproliferative effect of TNF-α has been demonstrated in various malignancies such as prostate (Sherwood et al, 1990), colon (Kemeny et al, 1990), (Heim et al, 1990), (Schiller et al, 1990), kidney carcinomas (Skillings et al, 1992), as well as malignant melanoma (Lienard et al, 1992). An antiproliferative effect of TNF-α has also been found in lung cancer cell lines where an

© 2000 Cancer Research Campaign antitumorigenic function of TNF-α has been observed (Hong et al, 1987; Munker et al, 1987; Yang et al, 1989). In vivo, Ohkura et al (1990) have reported an inhibitory effect in vivo of a combined TNF-α/IL1 treatment on pulmonary metastases in mice affected by lung carcinoma. Diffuse macrophage clustering and tumour hypoxia, frequently associated with lung cancer, may increase the level of TNF-α and/or modify the expression of TNF-α receptors, influencing the effect of this cytokine on tumour angiogenesis.

In the current study, coexpression of TNF-α, TNFR-I and TNFR-II in NSCLC has been found by RT-PCR assay, as also reported by Tran et al (1998) who used an immunohistochemical technique. The PCR method cannot discriminate between free TNF-α and receptor-bound TNF-α, so that the positive signal we found in our cases can either depend on TNF-α secreted by the tumour-infiltrating inflammatory cells, or result from autocrine production by the tumour cells. Further analyses using immunocytochemistry will be performed to localize TNF-α and their receptors, in order to clarify the paracrine or autocrine effect of this cytokine in NSCLC. The statistically significant association we found in our series of NSCLC between TNF-α and favourable prognosis may be related to the influence of TNF-α on tumour angiogenesis. In fact, tumours expressing TNF-α showed a number of microvessels significantly lower than tumours which did not express it.

Since our previous studies demonstrated a connection between NSCLC behaviour, neoangiogenesis and *bcl*-2 expression, we investigated the putative relation between TNF-α on the one hand, and vascular count (as a measure of tumour angiogenesis) and *bcl*-2 protein expression on the other. Our results show a significant direct association between TNF-α and *bcl*-2; moreover, as previously demonstrated, we observed an inverse significant correlation between *bcl*-2 protein expression and vascular count, suggesting that the favorable effect of TNF-α on clinical outcome may be related to a low *bcl*-2-mediated neo-vascular development. It has been experimentally demonstrated that *bcl*-2-expressing cells (Hennet et al, 1993) may survive to hypoxic alteration induced by TNF-α in mouse fibrosarcoid cells.

This putative *bcl*-2-dependent resistance in NSCLC cells could prevent necrosis and consequent hypoxic-induced angiogenic growth factor up-regulation. Interesting data in this sense have been reported in a series of NSCLC we previously analysed, which showed a significant association between *bcl*-2 expression and low levels of vascular endothelial growth factor. Hypoxic alterations determined by high levels of TNF-α, induced by the large amount of inflammatory cells frequently present in NSCLC, were able to produce necrotic alterations in a great number of neoplastic cells preserving *bcl*-2-expressing ones, with consequent reduced angiogenesis and better prognosis.

### TGF-β expression

The effects of TGF-β on tumour growth are controversial, so that TGF-β is able to directly inhibit proliferation of epithelial cancer cells (Lippman et al, 1987), but its immunosuppressive properties can also promote tumour growth. TGF-β activity is generally inhibitory to epithelial cells in vitro and has several effects on the regulation of extracellular matrix components (Sporn et al, 1986). Moreover, the cellular response to TGF-β and the presence of several isoforms could be important factors for explaining the conflicting results regarding its activity (Mizukami et al, 1990; McCune et al, 1992; Walker et al, 1992).

In our study no correlation was found between TGF-β mRNA expression and microvessels count, although samples with PCR-positive signal for TGF-β tended to have a lower number of vessels than the negative ones. However, we observed an interesting association between TGF-β expression and survival, since patients with TGF-β mRNA expression had a significantly better prognosis than patients who did not express this cytokine. Similar results were obtained also in breast carcinomas where the expression of TGF-β was associated with a longer disease-free interval (Murray et al, 1993) and with absence of nodal metastatic involvement. There is some evidence that the metastatic behaviour of a tumour is strongly determined by its ability to break down the basement membrane, possibly by the increased production of collagenase (Liotta et al, 1991). Some endogenous proteinase inhibitors may potentially inhibit this invasive process and the TGF-β expression may protect against invasion by locally regulating the basement membrane components from protease action.

These data can explain the association we found between TGF-β mRNA levels and better prognosis in our series of NSCLC, prompting us to further investigate the role of TGF-β as a prognostic indicator or as a potential target for innovative therapies in cancer.

### ACKNOWLEDGEMENTS

This work has been supported by grants from the Associazione Italiana per la Ricerca sul Cancro (AIRC).

### REFERENCES

American Joint Committee on Cancer (1992) *Manual for a staging of Cancer*, 4th ed: 115–122

Auvinen P, Lipponen P, Johansson R and Syrjanen K (1995) Prognostic significance of TGF-β1 and TGF-β2 expression in female breast cancer. *Anticancer Res* **15**: 2627–2632

Blood CH and Zetter BR (1990) Tumor interactions with the vasculature: angiogenesis and tumour metastasis. *Biochem Biophys Acta* **1032**: 89–118

Boldrini L, Calcinai A, Silvestri V, Basolo F, Lucchi M, Mussi A, Angeletti CA, Bevilacqua G and Fontanini G (1999) Quantitation by competitive PCR assay of vascular endothelial growth factor in non-small cell lung carcinomas. *International Journal of Oncology* **14**: 161–168

Cordell JL, Falini B, Erber WN, Abdulaziz Z, Macdonald S, Pulford KAF, Stein H and Mason DY (1984) Immunoenzymatic labelling of monoclonal antibodies using immune complexes of alkaline phosphatase and monoclonal anti-alkaline phosphatase (APAAP complex). *J Histochem Cytochem* **32**: 219–222

Derynk R, Jarrett JA, Chen EY, Eaton DH, Bell JR, Assoian RK, Roberts AB, Sporn NB and Baum M (1985) Human transforming growth factor-beta, complementary DNA sequence and expression in normal and transformed cells. *Nature* **316**: 701–705

Fajardo LF, Kwan HH, Kowalski J, Prionas SD and Allison AC (1992) Dual role of tumour necrosis factor a angiogenesis. *Am J Pathol* **140**: 539–544

Fajardo LF, Prionas SD, Kwan HH, Kowalski J and Allison AC (1996) Transforming growth factor b1 induces angiogenesis in vivo with a threshold pattern. *Lab Invest* **74**: 600–608

Fontanini G, Lucchi M, Vignati S, Mussi A, Ciardiello F, De Laurentiis M, De Placido S, Basolo F, Angeletti CA and Bevilacqua G (1997) Angiogenesis as a prognostic indicator of survival in non-small cell lung carcinoma: a prospective study. *J Natl Cancer Inst* **89**: 881–886

Fontanini G, Boldrini L, Vignati S, Chinè S, Basolo F, Silvestri V, Lucchi M, Mussi A, Angeletti CA, and Bevilacqua G (1998) Bcl-2 and p53 regulate vascular endothelial growth factor (VEGF)-mediated angiogenesis in non-small cell lung carcinoma. *Eur J Cancer* **34**: 718–723

Fox SB, Westwood M, Moghaddam A, Comley M, Turley H, Whitehouse RM, Bicknell R, Gatter K C and Harris AL (1996) The angiogenic factor platelet-derived endothelial cell growth factor/thymidine phosphorylase is up-regulated in breast cancer epithelium and endothelium. *Br J Cancer* **73**: 275–280

Hong WS, Jett JR, Sasaki Y, Takahashi H, Nakano H, Nakagawa K, Hoshi A and Saijo N (1987) Colony inhibitory effect of recombinant human tumour necrosis factor (rH-TNF) and/or recombinant human interferon (rH-IFN)-alpha, -beta, and -gamma on human lung cancer cell lines. *Jpn J Clin Oncol* **17**: 49–55

Kemeny N, Childs B, Larchian W, Rosado K and Kelsen D (1990) A phase II trial of recombinant tumour necrosis factor in patients with advanced colorectal carcinoma. *Cancer* **66**: 659–663

Koukourakis MI, Giatromanolaki A, O'Byrne KJ, Whitehouse RM, Talbot DC, Gatter KC and Adrian Harris (1997) Potential role of bcl-2 as a suppressor of tumour angiogenesis in non small-cell lung cancer. *Int J Cancer* **74**: 565–570

Heim ME, Siegmund R, Illiger HJ, Klee M, Rieche K, Berdel WE and Edler L (1990) Tumour necrosis factor in advanced colorectal cancer: a phase II study. A trial of phase I/II study group of the Association for Medical Oncology of the German Cancer Society. *Onkologie* **13**: 444–447

Hennet T, Bertoni G, Richter C and Peterhans E (1993) Expression of BCL-2 protein enhances the survival of Mouse fibrosarcoid cells in Tumour Necrosis Factor-mediated cytotoxicity. *Cancer Res* **53**: 1456–1460

Leek RD, Landers R, Fox SB, NGF, Harris AL and Lewis CE (1998) Association of tumour necrosis factor alpha and its receptors with thymidine phosphorylase expression in invasive breast carcinoma. *Br J Cancer* **77**: 2246–2251

Lewis CE and McGee JO'D (1996) The role of TNF-alpha: implications for prognosis and possible strategies for intervention. *Horizons in Medicine* **7**: 270–282

Lewis CE and Balkwill FR (1997) The complex molecular network regulating tumour angiogenesis. In *Tumour Angiogenesis*. Bicknell R, Lewis CE and Ferrara N (eds), pp. 111–113. Oxford University Press: Oxford

Lienard D, Lejeune FJ and Ewalenko P (1992) In transit metastases of malignant melanoma treated by high dose rTNF alpha in combination with interferon-gamma and melphalan in isolation perfusion. *World J Surg* **16**: 234–240

Liotta LA and Stetler-Stevenson WG (1991) Tumour invasion and metastasis: an imbalance of positive and negative regulation. *Cancer Res* **51**: 5054–5059

Lippman ME, Dickson RB, Gelmann EP, Rosen N, Knabbe C, Bates S, Bronzert D, Huff KE and Kasid A (1987) Growth regulation of human breast carcinoma occurs through regulated growth factor secretion. *J Cell Biochem* **35**: 1–16

McCune BK, Mullin BR, Flanders KC, Jaffers WS, Mullen LT and Sporn MB (1992) Localisation of transforming growth factor-beta isotypes in lesions of the human breast. *Hum Pathol* **23**: 13–20

Miles DW, Happerfield LC, Naylor MS, Bobrow LG, Rubens RD and Balkwill FR (1994) Expression of tumour necrosis factor (TNF alpha) and its receptors in benign and malignant breast tissue. *Int J Cancer* **56**: 777–782

Mizukami Y, Nonomura A, Yamada T, Kurumaya H, Hayashi M, Koyasaki N, Taniya T, Nonguchi M, Nakamura S and Matsubara F (1990) Immunohistochemical demonstration of growth factors TGF alpha, TGF beta, IFG-I and *neu* oncogene product in benign and malignant breast tissues. *Anticancer Res* **10**: 1115–1126

Munker M, Munker R, Saxton RS and Hoeffler HP (1987) Effect of recombinant monokines, lymphokines, and other agents on clonal proliferation of human cancer cell lines. *Cancer Res* **47**: 4081–4085

Murray PA, Barrett-Lee P, Travers M, Luqmani Y, Powels T and Coombes RC (1993) The prognostic significance of transforming growth factors in human breast cancer. *Br J Cancer* **67**: 1408–1412

Naylor MS, Stamp GW, Foulkes WD, Eccles D and Balkwill FR (1993) Tumour necrosis factor and its receptors in human ovarian cancer. Potential role in disease progression. *J Clin Invest* **91**: 2194–2206

Ohkura M, Fuchimoto S and Orita K (1990) Antitumour effect of recombinant human interleukin-1 beta alone and in combination with natural tumour necrosis factor-alpha. *Jpn J Cancer Res* **81**: 1026–1031

Pezzella F, Jones M, Ralfkier E, Ersbol J, Gatter KC and Mason DJ (1992) Evaluation of bcl2 protein expression and 14;18 translocation as prognostic markers in follicular lymphoma. *Br J Cancer* **65**: 87–89

Pezzella F, Turley H, Kuzu I, Tungekar MF, Dunnill MS, Pierce CB, Harris A, Gatter KC and Mason DY (1993) Bcl-2 protein in non small-cell lung carcinoma. *New Engl J Med* **329**: 690–694

Pusztai L, Clover LM, Cooper K, Starkey PM, Lewis CE and McGee JO'D (1994) Expression of tumour necrosis factor alpha and its receptors in carcinoma of the breast. *Br J Cancer* **70**: 289–292

Rubbert A, Manger B, Lang N, Kalden JR and Platzer E (1991) Functional characterization of tumour-infiltrating lymphocytes, lymphonode lymphocytes, and peripheral lymphocytes. *Int J Cancer* **49**: 25–31

Silvestrini R, Costa A, Lequaglie C, Mochen C, Veneroni S, Leutner M, and Ravasi G (1998) Bcl-2 protein and prognosis in patients with potentially curable non-small-cell lung cancer. *Virchows Arch* **432**: 441–444

Schiller JH and Bittner G (1990) Anti-proliferative effects of tumour necrosis factor, gamma interferon, and 5-fluorouracil on human colorectal carcinoma cell lines. *Int J Cancer* **46**: 61–66

Sporn MB, Roberts AB, Wakefield LM and Assoian RK (1986) Transforming growth factor beta: biological function and chemical structure. *Science* **233**: 532–534

Tran T-A, Kallakury BVS, Ambros RA and Ross JS (1998) Prognostic significance of tumour necrosis factors and their receptors in nonsmall cell lung carcinoma. *Cancer* **83**: 276–282

Ueki N, Nakazato M, Ohkawa T, Ikeda T, Amuro Y, Hada T and Higashino K (1992) Excessive production of TGFβ1 can play an important role in the development of tumorigenesis by its action for angiogenesis: validity of neutralizing antibodies to block tumour growth. *Biochem Biophys Acta* **1137**: 189–196

Walker RA and Dearing SJ (1992) Transforming growth factor $beta_1$ in ductal carcinoma *in situ* and invasive carcinomas of the breast. *Eur J Cancer* **28**: 641–644

World Health Organization (1982) The World Health Organization histological typing of lung tumours. *Am J Clin Pathol* **77**: 123–136

Yang SC, Owen-Schaub LB, Grimm EA and Roth JA (1989) Induction of lymphokine-activated killer cytotoxicity with interleukin-2 and tumour necrosis factor-alpha against primary lung cancer targets. *Cancer Immunol Immunother* **29**: 193–198

*© 2000 Cancer Research Campaign*

## References

[PDF_00571] Auvinen P., Lipponen P., Johansson R., Syrjänen K. (1995). Prognostic significance of TGF-beta 1 and TGF-beta 2 expressions in female breast cancer.. Anticancer Res.

[PDF_00574] Blood C. H., Zetter B. R. (1990). Tumor interactions with the vasculature: angiogenesis and tumor metastasis.. Biochim Biophys Acta.

[PDF_00576] Boldrini L., Calcinai A., Silvestri V., Basolo F., Lucchi M., Mussi A., Angeletti C. A., Bevilacqua G., Fontanini G. (1999). Quantitation by competitive PCR assay of vascular endothelial growth factor in non-small cell lung carcinomas.. Int J Oncol.

[PDF_00580] Cordell J. L., Falini B., Erber W. N., Ghosh A. K., Abdulaziz Z., MacDonald S., Pulford K. A., Stein H., Mason D. Y. (1984). Immunoenzymatic labeling of monoclonal antibodies using immune complexes of alkaline phosphatase and monoclonal anti-alkaline phosphatase (APAAP complexes).. J Histochem Cytochem.

[PDF_00585] Derynck R., Jarrett J. A., Chen E. Y., Eaton D. H., Bell J. R., Assoian R. K., Roberts A. B., Sporn M. B., Goeddel D. V. (1985). Human transforming growth factor-beta complementary DNA sequence and expression in normal and transformed cells.. Nature.

[PDF_00589] Fajardo L. F., Kwan H. H., Kowalski J., Prionas S. D., Allison A. C. (1992). Dual role of tumor necrosis factor-alpha in angiogenesis.. Am J Pathol.

[PDF_00591] Fajardo L. F., Prionas S. D., Kwan H. H., Kowalski J., Allison A. C. (1996). Transforming growth factor beta1 induces angiogenesis in vivo with a threshold pattern.. Lab Invest.

[PDF_00598] Fontanini G., Boldrini L., Vignati S., Chinè S., Basolo F., Silvestri V., Lucchi M., Mussi A., Angeletti C. A., Bevilacqua G. (1998). Bcl2 and p53 regulate vascular endothelial growth factor (VEGF)-mediated angiogenesis in non-small cell lung carcinoma.. Eur J Cancer.

[PDF_00594] Fontanini G., Lucchi M., Vignati S., Mussi A., Ciardiello F., De Laurentiis M., De Placido S., Basolo F., Angeletti C. A., Bevilacqua G. (1997). Angiogenesis as a prognostic indicator of survival in non-small-cell lung carcinoma: a prospective study.. J Natl Cancer Inst.

[PDF_00602] Fox S. B., Westwood M., Moghaddam A., Comley M., Turley H., Whitehouse R. M., Bicknell R., Gatter K. C., Harris A. L. (1996). The angiogenic factor platelet-derived endothelial cell growth factor/thymidine phosphorylase is up-regulated in breast cancer epithelium and endothelium.. Br J Cancer.

[PDF_00618] Heim M. E., Siegmund R., Illiger H. J., Klee M., Rieche K., Berdel W. E., Edler L. (1990). Tumor necrosis factor in advanced colorectal cancer: a phase II study. A trial of the phase I/II study group of the Association for Medical Oncology of the German Cancer Society.. Onkologie.

[PDF_00622] Hennet T., Bertoni G., Richter C., Peterhans E. (1993). Expression of BCL-2 protein enhances the survival of mouse fibrosarcoid cells in tumor necrosis factor-mediated cytotoxicity.. Cancer Res.

[PDF_00608] Hong W. S., Jett J. R., Sasaki Y., Takahashi H., Nakano H., Nakagawa K., Hoshi A., Saijo N. (1987). Colony inhibitory effect of recombinant human tumor necrosis factor (rH-TNF) and/or recombinant human interferon (rH-IFN)-alpha, -beta and -gamma on human lung cancer cell lines.. Jpn J Clin Oncol.

[PDF_00612] Kemeny N., Childs B., Larchian W., Rosado K., Kelsen D. (1990). A phase II trial of recombinant tumor necrosis factor in patients with advanced colorectal carcinoma.. Cancer.

[PDF_00615] Koukourakis M. I., Giatromanolaki A., O'Byrne K. J., Whitehouse R. M., Talbot D. C., Gatter K. C., Harris A. L. (1997). Potential role of bcl-2 as a suppressor of tumour angiogenesis in non-small-cell lung cancer.. Int J Cancer.

[PDF_00625] Leek R. D., Landers R., Fox S. B., Ng F., Harris A. L., Lewis C. E. (1998). Association of tumour necrosis factor alpha and its receptors with thymidine phosphorylase expression in invasive breast carcinoma.. Br J Cancer.

[PDF_00639] Lippman M. E., Dickson R. B., Gelmann E. P., Rosen N., Knabbe C., Bates S., Bronzert D., Huff K., Kasid A. (1987). Growth regulation of human breast carcinoma occurs through regulated growth factor secretion.. J Cell Biochem.

[PDF_00634] Liénard D., Lejeune F. J., Ewalenko P. (1992). In transit metastases of malignant melanoma treated by high dose rTNF alpha in combination with interferon-gamma and melphalan in isolation perfusion.. World J Surg.

[PDF_00642] McCune B. K., Mullin B. R., Flanders K. C., Jaffurs W. J., Mullen L. T., Sporn M. B. (1992). Localization of transforming growth factor-beta isotypes in lesions of the human breast.. Hum Pathol.

[PDF_00645] Miles D. W., Happerfield L. C., Naylor M. S., Bobrow L. G., Rubens R. D., Balkwill F. R. (1994). Expression of tumour necrosis factor (TNF alpha) and its receptors in benign and malignant breast tissue.. Int J Cancer.

[PDF_00648] Mizukami Y., Nonomura A., Yamada T., Kurumaya H., Hayashi M., Koyasaki N., Taniya T., Noguchi M., Nakamura S., Matsubara F. (1990). Immunohistochemical demonstration of growth factors, TGF-alpha, TGF-beta, IGF-I and neu oncogene product in benign and malignant human breast tissues.. Anticancer Res.

[PDF_00653] Munker M., Munker R., Saxton R. E., Koeffler H. P. (1987). Effect of recombinant monokines, lymphokines, and other agents on clonal proliferation of human lung cancer cell lines.. Cancer Res.

[PDF_00656] Murray P. A., Barrett-Lee P., Travers M., Luqmani Y., Powles T., Coombes R. C. (1993). The prognostic significance of transforming growth factors in human breast cancer.. Br J Cancer.

[PDF_00659] Naylor M. S., Stamp G. W., Foulkes W. D., Eccles D., Balkwill F. R. (1993). Tumor necrosis factor and its receptors in human ovarian cancer. Potential role in disease progression.. J Clin Invest.

[PDF_00662] Ohkura M., Fuchimoto S., Orita K. (1990). Antitumor effect of recombinant human interleukin-1 beta alone and in combination with natural human tumor necrosis factor-alpha.. Jpn J Cancer Res.

[PDF_00665] Pezzella F., Jones M., Ralfkiaer E., Ersbøll J., Gatter K. C., Mason D. Y. (1992). Evaluation of bcl-2 protein expression and 14;18 translocation as prognostic markers in follicular lymphoma.. Br J Cancer.

[PDF_00668] Pezzella F., Turley H., Kuzu I., Tungekar M. F., Dunnill M. S., Pierce C. B., Harris A., Gatter K. C., Mason D. Y. (1993). bcl-2 protein in non-small-cell lung carcinoma.. N Engl J Med.

[PDF_00671] Pusztai L., Clover L. M., Cooper K., Starkey P. M., Lewis C. E., McGee J. O. (1994). Expression of tumour necrosis factor alpha and its receptors in carcinoma of the breast.. Br J Cancer.

[PDF_00674] Rubbert A., Manger B., Lang N., Kalden J. R., Platzer E. (1991). Functional characterization of tumor-infiltrating lymphocytes, lymph-node lymphocytes and peripheral-blood lymphocytes from patients with breast cancer.. Int J Cancer.

[PDF_00680] Schiller J. H., Bittner G. (1990). Anti-proliferative effects of tumor necrosis factor, gamma interferon and 5-fluorouracil on human colorectal carcinoma cell lines.. Int J Cancer.

[PDF_00677] Silvestrini R., Costa A., Lequaglie C., Mochen C., Veneroni S., Leutner M., Ravasi G. (1998). Bcl-2 protein and prognosis in patients with potentially curable non-small-cell lung cancer.. Virchows Arch.

[PDF_00683] Sporn M. B., Roberts A. B., Wakefield L. M., Assoian R. K. (1986). Transforming growth factor-beta: biological function and chemical structure.. Science.

[PDF_00686] Tran T. A., Kallakury B. V., Ambros R. A., Ross J. S. (1998). Prognostic significance of tumor necrosis factors and their receptors in nonsmall cell lung carcinoma.. Cancer.

[PDF_00689] Ueki N., Nakazato M., Ohkawa T., Ikeda T., Amuro Y., Hada T., Higashino K. (1992). Excessive production of transforming growth-factor beta 1 can play an important role in the development of tumorigenesis by its action for angiogenesis: validity of neutralizing antibodies to block tumor growth.. Biochim Biophys Acta.

[PDF_00693] Walker R. A., Dearing S. J. (1992). Transforming growth factor beta 1 in ductal carcinoma in situ and invasive carcinomas of the breast.. Eur J Cancer.

[PDF_00699] Yang S. C., Owen-Schaub L., Grimm E. A., Roth J. A. (1989). Induction of lymphokine-activated killer cytotoxicity with interleukin-2 and tumor necrosis factor-alpha against primary lung cancer targets.. Cancer Immunol Immunother.

